# Spatial Relationships and Impact Effects between Urbanization and Ecosystem Health in Urban Agglomerations along the Belt and Road: A Case Study of the Guangdong-Hong Kong-Macao Greater Bay Area

**DOI:** 10.3390/ijerph192316053

**Published:** 2022-11-30

**Authors:** Yan Wu, Yingmei Wu, Chen Li, Binpin Gao, Kejun Zheng, Mengjiao Wang, Yuhong Deng, Xin Fan

**Affiliations:** 1 Faculty of Geography, Yunnan Normal University, Kunming 650500, China; 2 Yunnan Academy of Social Sciences, Kunming 650000, China; 3 College Humanities and Development Studies, China Agricultural University, Beijing 100193, China; 4 Center for Turkmenistan Studies, China University of Geosciences, Wuhan 430074, China

**Keywords:** bivariate spatial autocorrelation, ecosystem health, OPGD model, spatial regression, the Guangdong–Hong Kong–Macao Greater Bay Area (GBA), urbanization

## Abstract

A healthy ecosystem is fundamental for sustainable urban development. Rapid urbanization has altered landscape patterns and ecological functions, resulting in disturbances to ecosystem health. Exploring the effects of urbanization on ecosystem health and the spatial relationships between them is significant for cities along the “Belt and Road” aiming to achieve sustainable regional development. This study took the Guangdong–Hong Kong–Macao Greater Bay Area (GBA) as an example and measured the urbanization level (UL) and ecosystem health index (EHI) from 2000 to 2020 using multisource data. We used bivariate spatial autocorrelation, the geographically weighted regression model (GWR), and the optimal parameters-based geographical detector (OPGD) model to clarify the impact of urbanization on ecosystem health and the spatial relationship between them from multiple perspectives. The major findings of this study were: (1) the EHI in the GBA decreased significantly during the study period, dropping from 0.282 to 0.255, whereas the UL increased significantly, exhibiting opposite spatial distribution features; (2) there was a significant negative spatial correlation between UL and the EHI and significant spatial heterogeneity between high–low and low–high types in the GBA; (3) the negative effects of urbanization on ecosystem health were predominant and becoming more pronounced in the central GBA. Moreover, urbanization had an increasingly significant negative effect, leading to the deterioration of ecosystem health, in the central GBA. Population urbanization drove land urbanization, which became the main factor affecting ecosystem health in the GBA. Overall, urbanization had a significant negative effect on ecosystem health, with this impact being particularly prominent in the core urban junctions of the GBA, which require urgent attention. The results of the study provide a basis for decision making in the context of the steady urbanization and ecosystem health protection of cities along the “Belt and Road”.

## 1. Introduction

The concept of ecosystem health was introduced in the 1980s [1]. As one of the integrated characteristics of ecosystems, ecosystem health is closely related to the concept of sustainability and considered a concrete expression of the structural and functional integrity of ecosystems subject to disturbances by human activities, reflecting their stability and sustainability [2,3]. Healthy ecosystems provide required services to people and have great social and economic value [4]. In the past 40 years, ecosystem health has received increased attention from macroecologists worldwide. A wide variety of methods have been reported for measuring regional ecosystem health, such as the pressure–state–response (PSR) [5,6], vigor–organization–resilience (VOR) [7], and vigor–organization–resilience–services (VORS) models [8]. Accordingly, a series of studies on ecosystem health have been conducted in urban agglomerations [9], provincial urban areas [10], and watersheds [11]. Concomitantly, scholars have tried to incorporate ecosystem services into measurements of ecosystem health by constructing the vigor-organization-resilience-services (VORS) model [8], which effectively combines the health of the physical space of ecosystems with the human demand for ecosystem services to more comprehensively evaluate the state of spatial entities [12,13]. Notably, urban ecosystem health, an important monitoring indicator of sustainable development, provides the necessary conditions for human survival and development and has become the basis for regional sustainable development [14,15]. Currently, intensive human activities have significantly altered the structure and function of urban ecosystems, causing a spiraling decline in ecosystem health and, thus, posing a significant threat to the sustainability of human well-being [16]. Therefore, an integrated analysis of the health of urban ecosystems is essential to achieve sustainability.

Over the past four decades, China has undergone the largest process of urbanization in the world [17]. According to statistics, from 1978 to 2020, the urbanization rate in China increased from 17.9% to 63.89%, while the urban population increased from 170 million to 900 million in 2020, an increase of approximately 700 million people. Furthermore, the area of urban construction land increased from 0.67 million square kilometers in 1981 to approximately 133.7 thousand square kilometers in 2019 (a ~20-fold increase), and the number of cities has also grown from less than 200 to more than 600 [17]. While urbanization has provided people with improved, rich economic conditions and good coverage of material needs, on the other hand, as large numbers of people gather in concentrated areas, economic growth and urban expansion have affected land-use patterns and ecosystem functions, causing the degradation of ecosystems, exacerbating ecological risks, and seriously threatening the sustainability of ecosystem health [18,19,20]. With the growing concern for sustainable development and conservation of healthy ecosystems, numerous studies have been conducted on urbanization and ecosystem health from different perspectives. Li et al. used the coupling coordination degree model to measure the degree of coordination between urbanization and ecosystem health and attempted to explore the interaction mechanism between these factors using the GTWR model [21]. Zhang et al. discussed the means by which urbanization affected the health of Tibetan ecosystems based on topographic gradients and emphasized that topography had important implications for particular cities in terms of urbanization development and ecosystem health maintenance [22]. Cheng et al. tried to use spatial regression to analyze the mechanisms of urbanization and ecosystem health, taking the middle reaches of the Yangtze River urban agglomeration in China as an example, and found an obvious U-shaped curve relationship between them [23]. In addition, scholars have gradually started focusing on important discriminations in the effects of urbanization subsystem mechanisms on ecosystem health. Qiao et al. focused on land urbanization and found that it had a significant negative spatial spillover effect on ecosystem health [24]. Existing studies have shown that the interaction mechanisms between urbanization and ecosystem health are complex [21,23]. Therefore, considering the comprehensiveness and scientific standards required for such research, it is necessary to scientifically comprehend the spatial relationship between urbanization level (UL) and the ecosystem health index (EHI). Simultaneously, the implementation of relevant improvements complementary to the effects of urbanization subsystems on ecosystem health has become a prerequisite for achieving sustainable development and meeting the needs of human well-being.

Urban agglomerations are the most important geographical manifestations of urbanization and are associated with the most prominent contradictions between economic development and ecological protection and the most serious risks and challenges for sustainable development [25]. As one of the four major bay areas worldwide, the Guangdong–Hong Kong–Macao Greater Bay Area (GBA) is an important part in the construction of China’s “Belt and Road” plan and the main battlefield of new urbanization areas, as well as the most dynamic—and potentially the core—region for future economic development strategies and one of China’s modernization engines, undertaking the important task of the economic development of the country [26,27]. Existing studies have shown that rapid urbanization has not only crowded out a large area of ecological land in the GBA but also intensified the degree of habitat fragmentation [28], with the substantial degradation of the ecological landscape resulting in increasingly severe ecological and environmental issues in the area [29]. Moreover, the negative effects of urbanization, such as excessive population density and drastic changes in land-use patterns, have posed serious threats to the ecosystem environment [28,30]. Thus, numerous questions arise. In what ways are the UL and EHI affected during the process of high-speed and large-scale urbanization in the GBA? What is the spatial relationship between the UL and EHI? What are the effects of urbanization and urbanization subsystems on ecosystem health? These are important topics worthy of in-depth discussion and research.

The main objectives of this study included: (1) the measurement of the UL and EHI in the GBA through the construction of a comprehensive urbanization and ecosystem health assessment model; (2) the analysis of the spatial relationship between the UL and EHI in the GBA; and (3) the investigation of the impact effects of urbanization and urbanization subsystems on ecosystem health. Our study enriches the research on the spatial relationship between and impact effects of ULs and EHIs while providing a theoretical reference and practical basis for the coordinated promotion of urbanization and the scientific management and protection of ecosystem health in this world-class bay area.

## 2. Materials and Methods

### 2.1. Study Area

**Figure 1:**
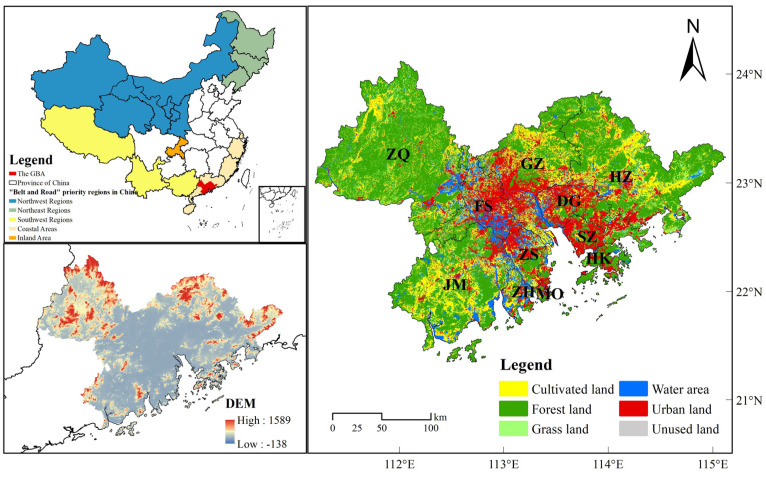
Location of the study area. Note: ZQ: Zhaoqing; FS: Foshan; JM: Jiangmen; ZS: Zhongshan; ZH: Zhuhai; GZ: Guangzhou; DG: Dongguan; SZ: Shenzhen; HZ: Huizhou; HK: Hongkong; MO: Macao.

The Guangdong–Hong Kong–Macao Greater Bay Area (GBA) is an important part of China’s “Belt and Road” coastal zone located in the Pearl River Delta region (21°25′–24°30′ N, 111°12′–115°35′ E) and includes nine cities (Guangzhou, Shenzhen, Zhuhai, Zhongshan, Jiangmen, Foshan, Dongguan, Huizhou, and Zhaoqing), as well as the two special administrative regions of Hong Kong and Macau (Figure 1). The overall climate is mainly a subtropical monsoon climate, warm and humid all year round, with an average annual temperature of 21–23 °C, average annual precipitation of over 1500 mm, and simultaneous occurrence of rain and heat. The vegetation type is mainly subtropical evergreen broad-leaved forests, and the terrain is mainly hilly. In 2020, the total economic volume of the GBA reached CNY 11.5 trillion, CNY 1.4 trillion more than in 2017, with the urbanization rate already exceeding 85%, ranking the area fifth in China. With less than 0.6% of the country’s area, the GBA generates approximately 13% of the country’s gross domestic product (GDP). As a pioneer area for deepening reform and progress in all aspects, including new-type urbanization, and a demonstration area for inland progression and cooperation, it occupies an important position in China’s regional development strategies. However, the high-speed urbanization and economic development have brought huge challenges to the construction of ecological civilization in the GBA.

### 2.2. Data Sources

The study data comprised two main types of data: physical geographical and socioeconomic data. Land-use data for 2000, 2010, and 2020 (with a spatial resolution of 30 m) were mainly interpreted from GlobeLand30, which is the world’s first global land cover product with a spatial resolution of 30 m [31]. We classified land types into six types: cultivated land, forest land, grassland, water area, urban land, and unused land. Table 1 shows the specific sources for each of these data types used in our study.

### 2.3. Research Framework and Methods

#### 2.3.1. Research Framework

**Figure 2:**
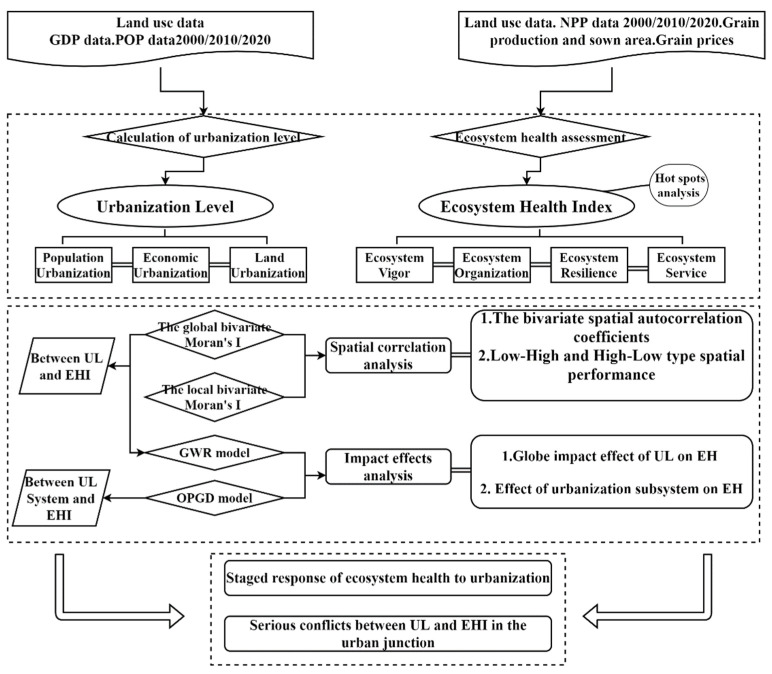
Research framework.

We constructed a comprehensive urbanization-level evaluation model with typical indicators of population, economy, and land urbanization and quantitatively analyzed the ecosystem health based on the VORS model. We determined the spatial relationship between urbanization and ecosystem health using bivariate spatial autocorrelation and, further, attempted to use geographically weighted regression (GWR) and an optimal parameters-based geographical detector (OPGD) model. We moreover explored the impact effects of urbanization and urbanization subsystems on ecosystem health in order to expand and improve our research results on the ecosystem health of urban agglomerations under the process of rapid urbanization and provide scientific references for realizing the conservation and utilization of the ecosystem in the GBA and promoting its sustainable economic and social development. We divided the study area into a 5 × 5 km grid, 2594 grids in total. The specific research framework is as follows (Figure 2).

#### 2.3.2. Ecosystem Health Assessment

In addition to the evaluation of the physical health of ecosystems, the ecosystem services capacity is also an important measure of ecosystem health for a comprehensive picture [3]. Based on existing studies, we attempted to build a comprehensive evaluation index system using the vigor-organization-resilience-services (VORS) model [22,32], which can be used to comprehensively measure the ecosystem health value of an area. The formulas used were as follows:

(1) $$\mathrm{EHI}=\sqrt{\mathrm{PHI}\times \mathrm{ES}}$$

(2) $$\mathrm{PHI}=\sqrt[{3}]{\mathrm{EV}\times \mathrm{EO}\times \mathrm{ER}}$$

where EHI indicates the ecosystem health index; PHI and ES represent the ecosystem physical health status and ecosystem services value, respectively; and EV, EO, and ER stand for ecosystem vigor, ecosystem organization, and ecosystem resilience, respectively. To reduce the various index dimensions, all indices were standardized from 0 to 1.

(1) Ecosystem Vigor (EV)

The vitality of an ecosystem indicates its metabolic capacity, which is an important expression of ecosystem function [3]. Net primary productivity (NPP) reflects the efficiency of plant fixation and conversion of photosynthetic products and is a useful indicator of the impact of urban development on regional logistics and energy flows that can also be used to assess the state of ecosystem vitality in a study area [32,33].

(2) Ecosystem Organization (EO)

Ecosystem organization refers to the organizing power of an ecosystem, which is reflected in structural stability and determined by landscape heterogeneity and connectivity [34]. In previous studies, the Shannon diversity index (SHDI) and area-weighted mean patch fractal dimension index (AWMPFD) have been proposed to characterize the spatial heterogeneity of a landscape; the landscape fragmentation index (FN_1_) and landscape sprawl index (CONT) have been proposed to characterize the connectivity of a whole landscape, whereas the habitat connectivity can be characterized by the fragmentation index and landscape sprawl index of woodlands, grasslands, and watersheds [12,33,35]. Weights were set according to the study by Peng et al. [35]. The formula used was as follows:

(3) $$\begin{array}{c} \mathrm{EO}=0.35\mathrm{LH}+0.35\mathrm{LC}+0.30\mathrm{IC}=(0.25\mathrm{SHDI}+0.10\mathrm{AWMPFD})\\ +\left(0.25{\mathrm{FN}}_{1}+0.10\mathrm{CONT}\right)+\left(0.07{\mathrm{FN}}_{2}+0.03{\mathrm{COHE}}_{1}+0.07{\mathrm{FN}}_{3}\right.\\ \left.+0.03{\mathrm{COHE}}_{2}+0.07{\mathrm{FN}}_{4}+0.03{\mathrm{COHE}}_{3}\right) \end{array}$$

where EO is the index of regional ecosystem organization; LH is the landscape heterogeneity index; LC is the landscape connectivity index; IC indicates the patch connectivity index of important ecosystems (forest, water, and grassland); SHDI represents Shannon’s diversity index; AWMPFD refers to the area-weighted mean patch fractal dimension index; FN_1_ indicates the landscape fragmentation index; CONTAG is the landscape contagion index; and FN_2_, FN_3_, FN_4_, COHESION1, COHESION2, and COHESION3 denote the fragmentation indices and patch cohesion indices of forest, water, and grassland, respectively. All of the above landscape indices were available through the FRAGSTATS 4.2 platform.

(3) Ecosystem Resilience (ER)

Ecosystem resilience refers to the ability of a natural ecosystem to recover its original structure and function after external disturbances [3]. Due to the important role of land use in the concept of ecosystem resilience, He et al. [12,35] proposed resilience coefficients for different land-use types and calculated the resilience of different land types. Table 2 shows the ecosystem resilience coefficient for each of the land-use types in the GBA. The formula used was as follows:

(4) $$\mathrm{ER}=\sum_{i=1}^{n}A_{i}\times {\mathrm{ERC}}_{i}$$

where ER stands for ecosystem resilience; A_i_ denotes the area ratio of land-use type i; ERC_i_ denotes the ecosystem resilience coefficient of land-use type i; and n is the number of land-use types

(4) Ecosystem Service (ES)

The monetized ecosystem services value (ESV) is used to quantify the value of ecosystem services [22]. We comprehensively calculated the ESV in the GBA based on the study by Xie et al. and related studies in the region [36,37]. More specifically, we obtained the market price of grain yields from the Guangdong Provincial Statistical Yearbook and other sources and calculated the ESV per unit area of arable land as one seventh of the average grain-yield market economic value. Subsequently, we corrected the ESV per unit area using a biomass factor coefficient of 1.17 to derive an economic value of CNY 2204/hm^2^ for a single ecosystem service-equivalent factor in the GBA. Similarly, the value of various ecosystem services per unit area was obtained by converting different land-use types. The ESVs of different ecological system units (Table 3) were calculated according to the following formula:

(5) $$\mathrm{ESV}=\sum A_{k}\times V_{C}k$$

where ESV is the ecosystem service value; A_k_ is the area of land-use type k (hm^2^); and V_C_k is the ecological coefficient per unit area of land-use type k.

#### 2.3.3. Calculation of Urbanization Level

Urbanization is a complex system that includes the population urbanization subsystem, economic urbanization, land urbanization subsystems, etc. Bias towards any of these aspects reduces the comprehensive utility of urbanization [23,38]. As social data are difficult to spatialize, GDPD and POPD were used to quantify economic and demographic urbanization, while the proportion of built-up land (ULP) was used to represent land urbanization, and these measures were integrated into a composite indicator for the urbanization level (UL) [23,39]. As the spatial distributions of POPMD, GDPD, and ULP were highly similar, these three indicators were standardized for analysis. The formula for calculating the UL was as follows:

(6) $$\mathrm{UL}=\frac{U_{\mathrm{POPD}}^{\prime }+U_{\mathrm{GDPD}}^{\prime }+U_{\mathrm{ULP}}^{\prime }}{3}$$

where U’_POPD_, U’_GDPD_, and U’_ULP_ represent the standardized value of the POPD, GDPD, and ULP in the grid and UL represents the urbanization level in the grid.

#### 2.3.4. Hotspot Analysis

Hotspot analysis was used to identify areas of concentration for specific variables, and it is widely used in various research areas to explore the clustering of high or low ecosystem health values in space [40,41]. In spatial statistics, a hotspot analysis tool based on Gi*statistics (Getis-Ord Gi*statistics) in ArcGIS is usually used to analyze the cold-spot areas of ecosystem health [41].

#### 2.3.5. Spatial Correlation Analysis

Spatial autocorrelation refers to the statistical correlation between the value of an attribute of a geographic object and the difference in spatial location and is an important indicator of the aggregation or discrete distribution of spatial elements; it is generally described by global Moran’s I and local Moran’s I values [42]. To determine the spatial correlation between UL and the EHI, the global bivariate Moran’s I was used to examine the correlation between UL and EHI and its significance level from the global perspective. The local bivariate Moran’s I was then used to identify spatial autocorrelation patterns and local spatial instability. According to the local Moran’s I index, a high–high type in the calculation result indicates high urbanization and a high ecosystem health-type area, whereas a low–low type indicates low urbanization and a low ecosystem health-type area; likewise, a high–low type indicates high urbanization and a low ecosystem health-type area, whereas a low–high type indicates low urbanization and a high ecosystem health-type area [23,43]. The formula for calculating Moran’s I was as follows:

(7) $$\;{\mathrm{Moran}}^{\prime }s\;I=\frac{n}{\sum_{i=1}^{n}\sum_{j=1}^{n}W_{ij}}\times \frac{\sum_{i=1}^{n}\sum_{j=1}^{n}W_{ij}\left(x_{i}-\bar{x}\right)\left(x_{j}-\bar{x}\right)}{\sum_{i=1}^{n}{\left(x_{i}-\bar{x}\right)}^{2}}$$

where n is the number of research units; W_ij_ is the spatial weights matrix; X_i_ and X_j_ are the UL and EHI of units i and j, respectively; and x is the average of the EHI. The values of Moran’s I range from −1 to 1. Values <0, >0, and equal to 0 indicate negative spatial autocorrelation, positive spatial autocorrelation, and no spatial autocorrelation, respectively. The higher the Moran’s I absolute value is, the stronger the spatial autocorrelation.

(8) $$I_{\mathrm{kl}}^{i}=z_{k}^{i}\sum_{j=1}^{n}w_{\mathrm{ij}}z_{l}^{j}$$

where $z_{k}^{i}=\frac{X_{k}^{i}-\bar{X_{k}}}{e_{k}}$ and $z_{l}^{j}=\frac{X_{l}^{i}-\bar{X_{l}}}{e_{l}}$; $X_{k}^{i}$ is the value of attribute k of sampling plot i; $X_{l}^{j}$ is the value of attribute l of sampling plot j; $\bar{X_{k}}$ and $\bar{X_{l}}$ are the average values of attributes k and l, respectively; and e_k_ and e_l_ are the variances of attributes k and l, respectively.

#### 2.3.6. Impact Effects Analysis

(1) Spatial regression models

A geographically weighted regression model (GWR), which is a local regression model, is a spatial analysis technique for estimating parameters based on traditional regression models (OLS) that enables the direct simulation of the non-stationarity of different spaces and reflects the degree of influence of different geographical location variables on a region, thus complementing neglected local characteristics [44]. We explored the spatial heterogeneity of urbanization impacts on ecosystem health from the global perspective by using a GWR model. The formula was as follows:

(9) $$Y_{i}=\beta_{0}(\mu_{i},v_{i})+\sum_{k}\beta_{k}(\mu_{i},v_{i})X_{\mathrm{ik}}+\varepsilon_{i}$$

where Y_i_ is the dependent variable; X_ik_ is the explanatory variable of an n × n matrix; (μ_i_, v_i_) is a function of location; β_0_ is the constant term of regression; β_k_ is the parameter vector to be estimated; and ε_i_ is the random error term.

(2) Optimal parameters-based geographical detector model

Compared with traditional regression models, geographic detectors not only avoid multicollinearity but also reflect the driving effect of independent variables on dependent variables more strongly from the perspective of spatial heterogeneity [45]. We used the Optimal Parameters-based Geographical Detector (OPGD) model in the Factor Detection and Interaction Detection panels in R4.2 software (https://www.r-project.org, accessed on 20 October 2022) to explore the extent to which the urbanization subsystems affect ecosystem health. By comparing the effects of different dispersion methods and the number of stratification levels on the degree of factor interpretation, the model selects the optimal combination of dispersion methods for each factor, which helps to further improve the accuracy of the analysis results and to comprehensively reflect the effects of urbanization subsystems on ecosystem health [46,47].

## 3. Results

### 3.1. Spatiotemporal Characteristics of the UL and EHI in the GBA

#### 3.1.1. Spatiotemporal Characteristics of UL

**Figure 3:**
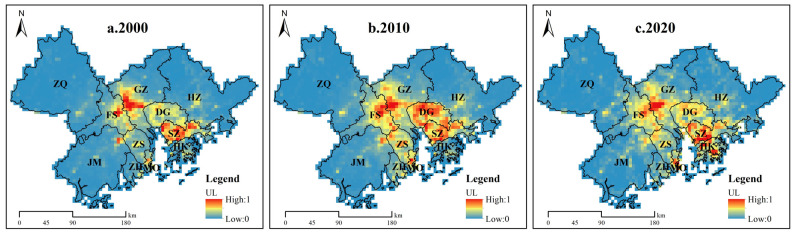
The spatial pattern of the UL in the GBA, 2000–2020.

Figure 3 depicts the spatial differences in the UL in the GBA from 2000 to 2020. We observed that areas with high ULs were concentrated in the central part of the GBA, with Guangzhou and Shenzhen being the leading “twin cores”. We identified a slight upward trend for the UL from 2000 to 2020, as indicated by the high-value areas at the junction of the core cities in the central part of the GBA; namely, Guangzhou–Foshan, Guangzhou–Dongguan–Shenzhen, Foshan–Dongguan, and Shenzhen–Hong Kong. These results indicated that, in the process of urbanization, development tends to always spread outward from the urban center, with the interaction between cities increasing and comprehensive agglomerations of higher-urbanization-level development forming at urban junctions.

#### 3.1.2. Spatiotemporal Characteristics of the EHI

We found that the average EHI values for the GBA from 2000 to 2020 were 0.282 (2000), 0.271 (2010), and 0.255 (2020), indicating an overall decreasing trend. We classified the EHI for the GBA into five classes: weak (0–0.08), relatively weak (0.08–0.18), ordinary (0.18–0.28), relatively good (0.28–0.38), and good (>0.38).

Table 4 exhibits the changes in the EHI in the study area from 2000 to 2020. We found that the percentages of areas with relatively good and good EHI values in 2000 accounted for 35.9% and 26.86% of the region, respectively. Interestingly, we observed that the areas with relatively good ecosystem health started to expand from 2000 and accounted for 48.93% by 2020, an increase of 13.03% from 2000. In contrast, the proportion of areas with good EHI decreased by 18.27%, dropping to only 8.59% in 2020. Moreover, the proportions of areas with weak and relatively weak ecosystem health increased by 2.48% and 2.72%, respectively. Overall, these findings indicated a significant overall decline in ecosystem health in the GBA.

**Figure 4:**
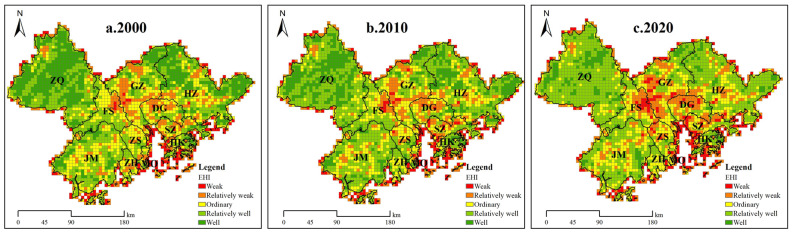
The spatial pattern of the EHI in the GBA, 2000–2020.

Figure 4 shows that the EHI in the GBA exhibited significant spatial heterogeneity and uneven spatial distribution. It is noteworthy that the areas with weak and relatively weak EHI values were mainly distributed in the central part of the GBA, overlapping with the distribution of regions with high-value urbanization. The central region of the GBA is conducive to urban development due to its relatively flat topography, relatively uniform land use, and the advantage of the bay, resulting in the largest distribution of urban junction areas with low ecosystem health values. For example, the Guangzhou–Foshan and Guangzhou–Dongguan–Shenzhen urban junctions belong to the core area of rapid economic development and, therefore, in this part of the region, the areas with weak and relative weak EHI were larger and showed further increases by 2020, indicating a concentrated, contiguous spatial distribution. Moreover, we noticed that the areas with good EHI values in 2000 were largely distributed in Zhaoqing and north of Huizhou but, by 2020, ecosystem health was affected by various factors and these areas were reclassified as areas with relatively good EHI.

**Figure 5:**
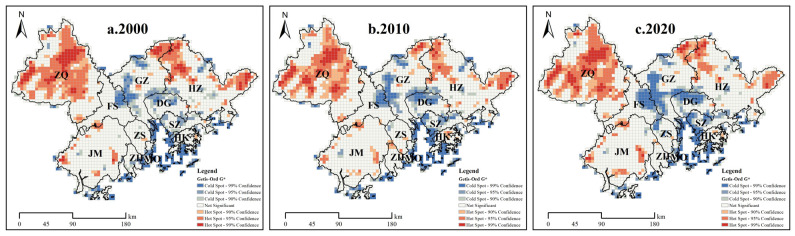
Spatial agglomeration characteristics of the EHI in the GBA, 2000–2020.

Based on hotspot analysis, we further investigated the spatial agglomeration characteristics and evolution of the EHI in the GBA from 2000 to 2020 (Figure 5). Specifically, we detected that the cold spot area was small but the distribution range was large and mainly concentrated in the central part of the GBA, including Guangzhou, Dongguan, Shenzhen, Foshan, Hong Kong, Macau, and other places, simultaneously indicating an expanding trend for urban junction areas in the central part of the GBA. In contrast, we found that hotspot areas were mainly located in Zhaoqing and Huizhou. Compared with the core cities in the central region of the GBA, these areas exhibited relatively low levels of urbanization development and, due to their hilly location and relatively dense forests and grasslands, the ecosystem health was relatively well maintained; however, it is necessary to be alert to disturbances of ecosystems caused by the outward thrust of urbanization.

### 3.2. Spatial Relationships between the UL and EHI in the GBA

**Figure 6:**
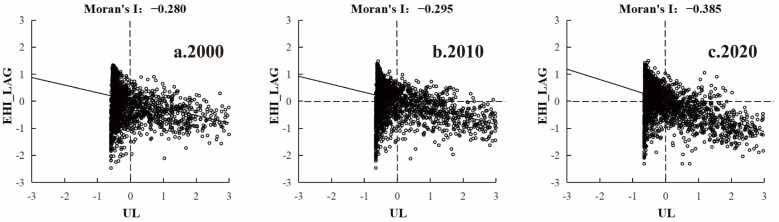
Global Moran’s I between the UL and EHI in the GBA, 2000–2020.

We found that the bivariate spatial autocorrelation coefficients for the UL and EHI of the GBA in 2000, 2010, and 2020 were −2.80, −2.95, and −3.85, respectively, with most values located in the second and fourth quadrants (Figure 6). The spatial relationship between the UL and EHI showed a gradually significant negative correlation, indicating that an increase in the value of the UL not only led to a decrease in the EHI in the immediate area but to an overall decrease in the surrounding areas.

**Figure 7:**
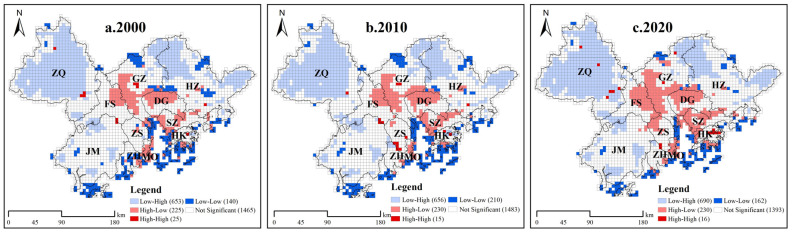
LISA cluster map of the UL and EHI in the GBA, 2000–2020.

To further analyze the spatial relationship between the UL and EHI of neighboring areas, we calculated the bivariate LISA using GeoDa1.14.0 software. We accordingly detected significant spatial heterogeneity (*p* < 0.05) in the spatial relationship between the UL and EHI in the GBA. In particular, we identified very prominent low–high- and high–low-type spatial performances, among which the low–high type covered the largest area distributed in a row north of Zhaoqing and Huizhou. Combining the urbanization development and the levels of ecosystem health of Zhaoqing and Huizhou, we observed that the development of urbanization levels in these two places was relatively low and associated with a relatively small impact on ecosystem health, thus highlighting the main clustering characteristics of low urbanization and high ecosystem health. We also noticed that, due to the spatial proximity effect, the high–low types were closely gathered and gradually formed a clumped distribution pattern. This suggested that the spatial gathering of high–low types was significant and the impact on the health of the ecosystem in the region was greater due to the rapid economic development and fast growth in urbanization in the GBA in the past two decades (Figure 7).

### 3.3. Impact Effect of UL on EH in the GBA

#### 3.3.1. Global Impact Effect of UL on EH

Spatial spillover effects require consideration when investigating the impact of UL on EH. Using a geographically weighted regression model (GWR), we analyzed the spatial heterogeneity in the impact of urbanization on ecosystem health. Table 5 lists the parameters in the two GWR models, showing that the goodness-of-fit values of the models were all above 0.5.

**Figure 8:**
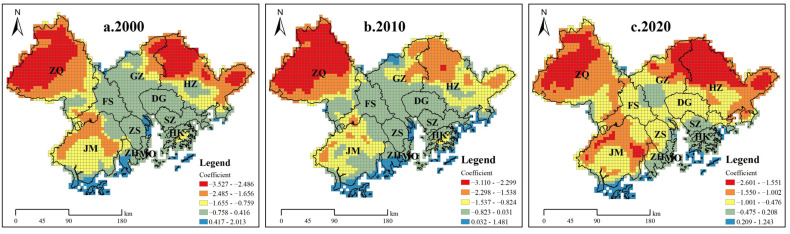
Spatial distribution of regression coefficients for the UL and EHI, 2000–2020.

We found that the overall values of the regression coefficients for urbanization and ecosystem health in the GBA were predominantly negative and dominated by negative effects, indicating that increased urbanization caused increased ecological pressure and drastically disturbed the ecosystem health (Figure 8). In particular, we noticed that the values of the coefficient changed dramatically with increasing urbanization, resulting in considerable pressure on the local ecological environment.

Regarding spatial distribution, the areas with high absolute values for the regression coefficients were concentrated to the north of Zhaoqing and Huizhou, indicating that the effect of urbanization on ecosystem health was weak; however, by 2020, the absolute values of the regression coefficients had increased, indicating a heightening of the degree to which urbanization impacted ecosystem health in this area. Moreover, we observed that the regions where the absolute value of the regression coefficient increased were concentrated in Guangzhou, Shenzhen, Foshan, Dongguan, Zhongshan, and Macau and Hong Kong in the central part of the GBA. Overall, we concluded that the degree of urbanization in the central part of the GBA had intensified, significantly affecting ecosystem health and resulting in prominent destruction of the ecosystem.

#### 3.3.2. Effect of Urbanization Subsystem on Ecosystem Health

Starting from the three subsystems of urbanization—POPD(X_1_), GDPD(X_2_), and ULP(X_3_)—we applied the OPGD model, emphasizing spatial data discretization methods and spatial layers, to detect the factors and their interactions affecting ecosystem health in the GBA from 2000 to 2020 (Table 6). We found that the overall ecosystem health of the GBA was strongly driven by POPD(X_1_) during the study period, with all q-values being above 0.3. With the rapid urbanization and the continuous expansion of construction land, we noticed the gradual emergence of the impact of ULP(X_3_) on ecosystem health, which, by 2020, had become the most important urbanization factor affecting ecosystem health with a q-value of 0.342. All q-values of POPD(X_1_) and GDPD(X_2_) showed a decreasing trend in the urbanization subsystem.

**Figure 9:**
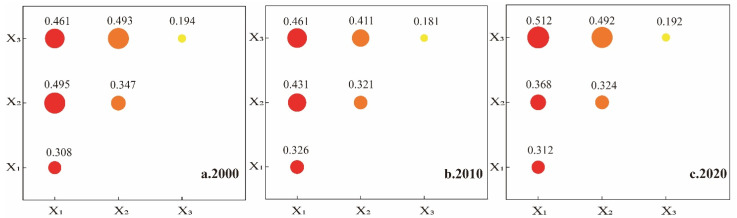
Detection results for the impact of the interaction of urbanization subsystems on ecosystem health, 2000–2020.

The results of the interaction detector module showed that any two of the drivers could be bifactor-enhanced without independence or weakening, indicating that the ecosystem health of the GBA was not the result of the action of a single urbanization factor but of a combination (Figure 9). In general, we detected that the combined interaction of population and land urbanization had a very strong impact on ecosystem health (X_1_ ∩ X_3_ interaction values were all above 0.4). From 2000 to 2010, the interaction values of X_1_ ∩ X_3_ were high, with continued momentum for population and economic urbanization and the influx into cities attracting large numbers of people, drastically affecting the stability of ecosystem health in the GBA. To meet the production and living needs of urban agglomerations, construction land rapidly expanded, resulting in a significant increase in the interaction value of X_1_ ∩ X_3_ after 2010 that reached its maximum in 2020 with an interaction value of 0.512. With the increasing interaction of population and land urbanization, the demand for construction land became greater, prompting its continuous expansion. In this process, the intense human activities in the GBA strongly disturbed the stability of the natural ecosystem, causing significant impacts on ecosystem health.

## 4. Discussion

### 4.1. Staged Response of Ecosystem Health to Urbanization

Scientific identification of the spatial and temporal evolution of ecosystem health and an accurate understanding of its dynamic relationship with the urbanization process are the keys to the harmonious development of megacities and environmental protection [48,49]. They are also outstandingly significant for cities along the “Belt and Road” aiming to steadily promote urbanization, maintain ecosystem stability and enhance human welfare. We introduced a GWR model to analyze the impact of urbanization on ecosystem health in the GBA and observed that the negative impact of urbanization was concentrated in the central part of the GBA and associated with varying differentiation characteristics. We aimed to identify which specific impact factors of urbanization were dominant in different periods. We further analyzed the extent of the impacts of population, economic, and land urbanization on ecosystem health and combined urbanization and ecosystem spatial relationships to identify the characteristics of the response of ecosystem health to urbanization.

In the early stages of urbanization (2000), the impact of population urbanization on ecosystem health was the most prominent (q = 0.331). This was due to the population-siphoning effect, which brought increasing numbers of people from the countryside to the cities and towns, with the related intense human activities stimulating the rapid development of urbanization and leading to the gradual threatening of ecosystem health. In this process, the proportion of the low–high-type spatial distribution was 25.17%, whereas that of the high–low-type was the smallest in the whole study period at only 8.67%, further indicating the prominent disturbance to ecosystem health in the early stages of urbanization (Table 7). Progressively, by 2010, the advantage of the GBA’s excellent location and the support of China’s national economic development strategy brought the rapid development of domestic and international cooperation and an influx of a large number of people, creating high economic value that led to the rapid urban development of core cities in the central part of the GBA. During this process, population and economic urbanization had strong impacts on ecosystem health, with q-values above 0.3. This rapid urbanization was accompanied by a significant impact on the low–low type ratio, which decreased by 1.16%. The Matthew effect in population and economic urbanization resulted in increasing numbers of people and economic factors gathering at the GBA. To meet the requirements of urban living and production, the proliferation of construction land became an important aspect of urbanization to a certain extent. Notably, population urbanization is known to drive land urbanization significantly, with an increase in the proportion of construction land playing a non-negligible role in the urbanization process [24,50]. In fact, urban land expanding faster than population growth is not a contradiction unique to China but a common phenomenon experienced as part of global urban development [51,52]. At the transition stage of urbanization development (2020), the role of economic urbanization in the impact on ecosystem health weakened due to the gradual transition of economic activities to green development following the realization that the rapid increase in construction land in the central part of the GBA was one of the main factors of ecosystem disturbance. With the promulgation of a series of measures for the implementation of regional cooperation, such as the “Key Action Plan for the Construction of Livable Bay Area around the Pearl River Delta” and the “Outline of the Development Plan of Guangdong, Hong Kong and Macao Greater Bay Area”, ecologically friendly living was vigorously promoted and the concept of green development was established, with this becoming the main theme of urbanization in the GBA. In this process, the proportion of low–high-type areas reached a 20 year maximum in 2020 at 26.60%, further confirming a stage of transition in the urbanization process towards development quality, livable conditions, and green protection that was intended to gradually reduce the crude impact of rapid urbanization on ecosystem health. As the trend towards the de-agriculturalization of the population is irreversible, it is necessary, with the premise of not affecting the living standards of urban residents, to pay further attention to the issue of construction land expansion and development patterns in the future promotion of urbanization. To this end, we recommended implementing effective measures to strictly control the scale of construction land, strengthening land macro-control, emphasizing the protection and improving the intensive use of land, ameliorating the threat to ecosystem health caused by the rapid urbanization of land, and ensuring efficient scientific management of urban construction land expansion.

### 4.2. Serious Conflicts between the UL and EHI in the Urban Junction

**Figure 10:**
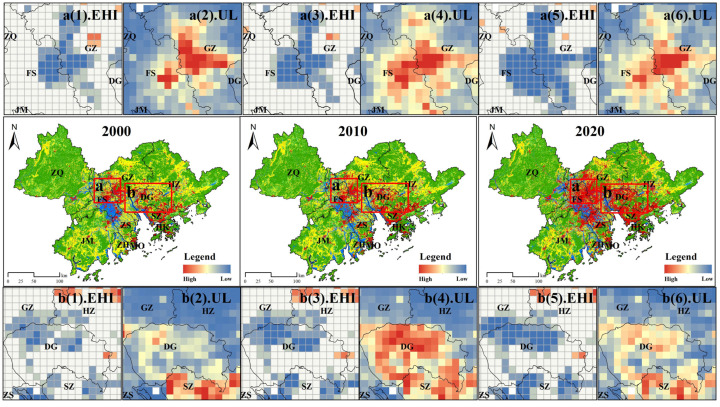
Changes in the EHI and UL in the core urban junction in the GBA, 2000–2020. Note: **a**(**1**)–**a**(**6**), distribution of EHI and ULs cold spots in a regions, 2000–2020; **b**(**1**)–**b**(**6**) distribution of EHI and ULs cold spots in b regions, 2000–2020.

Cultivation and growth is a historical process in the bay area. In the early stages of urbanization development, due to the influence of topographical factors, distance, and traffic, the cost of connection and communication between cities was high, resulting in loose connectivity between cities [53]. During the process of urbanization, with the gradual establishment of regional economic integration, the “push and pull” between cities compacted the original internal spatial structure and the construction of transportation networks and facilities broke the “distance boundary” between cities, with the radiation-driven effect between cities and urban clusters leading to the strengthening of internal ties and the establishment of urban junctions as the most directly and strongly interactive influence areas. This can be clearly observed for Guangzhou–Foshan (a) and Guangzhou–Dongguan–Shenzhen (b) in Figure 10. Regions a and b belong to the core urban junction in the central GBA, where spatial variations in urbanization and ecosystem health levels were particularly evident during the study period. In zone a (Guangzhou–Foshan), the Guangzhou–Foshan ecosystem health cold-spot zone expanded along the urban junction, with the urbanization level remaining at a high level during the same period. The change in the ecosystem health cold-spot area in zone b (Guangzhou–Dongguan–Shenzhen) is equally obvious. Among these areas, Dongguan has an excellent location advantage, with the capital city of Guangdong province (Guangzhou) in the north and the first demonstration area of China’s reform and opening up (Shenzhen) in the south, and the regional integration strategy in this area has driven the rapid development of urbanization. As an important node city in the Guangzhou–Shenzhen economic corridor, Dongguan was able to simultaneously take over the industries and economic pressure transferred from Guangzhou and Shenzhen, as well as a large number of people, thus increasing the overall urbanization level of the district while retaining remarkable ecosystem health. The areas with significant changes were at the urban interface of Guangzhou–Shenzhen, with the changes in town level and ecosystem health in Dongguan city center not being very prominent. This was mainly due to the fact that Dongguan, where urbanization is relatively well-established, has vigorously promoted the construction of green areas and parks and significantly improved the vitality of NPP ecosystems, improving the overall ecosystem health. In contrast, the urban junction exhibited relatively poor ecosystem fragmentation and resilience due to the urban fringe effect, contributing to the serious deterioration of ecosystem health. In conclusion, the core cities in the central part of the GBA have become increasingly closely connected and the "less urbanized" areas, which were originally on the peripheries of cities, have become more urbanized with the continuous improvements in transportation and policies. Therefore, paying attention to the ecosystem problems at urban junctions, steadily promoting urbanization, and strengthening the development and implementation of regional coordination and governance programs to avoid the emergence of “ecological and environmental protection gray areas” have become important aspects that need extra focus in the next step in urbanization in the GBA. Combined with the Guangdong Territorial Spatial Planning (2020–2035) strategy, the next step is to promote the linkage between the “twin cities” of Guangzhou and Shenzhen and the strong alliance between Guangzhou–Foshan and Hong Kong–Shenzhen cities and to support the formation of a clustered, multicenter, networking spatial pattern. In this process, we emphasize the need for “ecological priority and green development” and for a focus on the sustainable development of cities in the GBA and recommend establishing the idea of regional economic development integration, promoting the integration of ecological protection, and breaking the barriers to regional economic development while gradually improving the mechanisms linking ecological environmental protection among cities.

### 4.3. Limitations and Future Research

The use of bivariate spatial autocorrelation or spatial regression models to identify the spatial relationship between urbanization and ecosystem health has become a common research method [23,24], and a large number of studies have fully demonstrated that the relationship between urbanization and ecosystem health is not a simple linear one; however, the mechanism of feedback between them requires further exploration [54]. Considering that urbanization also has an impact on ecosystem health in surrounding areas, we attempted to combine the GWR and OPGD models to conduct a comprehensive analysis of the effects of urbanization on ecosystem health in terms of the overall level of urbanization and with different dimensions of urbanization represented by subsystems. This made it possible to more effectively reveal the spatial differentiation characteristics of urbanization and ecosystem health, demonstrating the degree to which urbanization subsystems impact ecosystem health at different stages and paving the way for subsequent research on urbanization development and ecosystem health protection in urban clusters. With the rapid advancement of urbanization, traffic accessibility [55] and the degree of POI aggregation [56] have gradually become important characterizing factors for the level of urbanization. In addition, as ecosystem health is not only affected by urbanization, we should also take into consideration other factors, such as climate change and PM2.5 emissions [32]. To further explore the factors influencing ecosystem health in the GBA and reveal the spatial relationships between different elements and ecosystem health, as well as the underlying mechanisms, a comprehensive analysis using multiple elements should be performed, and this would also provide a reasonable basis for relevant decisions.

Furthermore, the GBA is an important part of “the Belt and Road”, and the urbanization development and ecosystem health changes in the past two decades have highlighted different spatial relationships in different periods. Considering that most of the cities along the “Belt and Road” are at the stage of rapid urbanization development and there are large differences in urbanization development among the countries along the Belt and Road, promoting cooperation in urbanization is not only an inherent requirement to enhance the well-being of people in each country but also represents huge market potential. In the future, it will be necessary to grasp the dynamic relationships between urbanization and ecosystems in different time periods during the process of urbanization in countries along the Belt and Road, emphasizing ”ecological priority” and focusing on the core city junctions to avoid huge disturbances to the ecosystem as a result of urbanization while facilitating the radiating drive from core cities. We will strengthen the communication between cities along the “Belt and Road” and contribute to the modernization and prosperity of the people together.

## 5. Conclusions

Using multisource data, this study aimed to explore the spatial relationship between urbanization and ecosystem health, taking the Guangdong–Hong Kong–Macao Greater Bay Area as an example. We applied a global perspective using a GWR model to explain the spatial impact effects of urbanization on ecosystem health and supplemented the local perspective with an OPGD model to show the different levels at which urbanization subsystems impacted ecosystem health. The question regarding the effect of urbanization on ecosystem health was fully answered. Our methodology further emphasized the process of interaction between urbanization and ecosystems. It also highlighted significant implications for the coordinated development of urbanization and maintenance of ecosystem health in cities along the “Belt and Road”. Our conclusions were the following:

(1) We observed the rapid development of urbanization in the GBA from 2000 to 2020, with intense urbanization concentrated in the central part of the GBA. It was accompanied by an overall declining trend for ecosystem health, significant spatial differentiation of ecosystem health cold spots, and spatial dislocation characteristics in areas with high urbanization levels. These observations have a certain forward-looking significance for the development of urbanization along the “Belt and Road”. We recommend focusing on the coordination of urbanization development and ecosystem health, avoiding overly rapid urbanization development that could affect urban ecosystem health, and maintaining a positive overall level of ecosystem health;
(2) The spatial relationship between urbanization and ecosystem health showed a negative correlation characterized by high–low- and low–high-type spatial distributions, with large areas of high–low types being concentrated in the central part of the GBA and low–high types being concentrated in areas such as Zhaoqing and north of Huizhou, where urbanization is relatively weak. For the region along the “Belt and Road”, the rapid urbanization within cities and the pressure on the ecological environment require attention to be given to the coordinated development of the land structure. In addition, it is crucial to protect the ecosystems around the cities and allow them to perform their important ecological functions;
(3) The impact of urbanization on ecosystem health was mainly negative, with the regions with obvious and increasingly serious negative effects being concentrated in the central part of the country. The three urbanization subsystems had different levels of explanatory power with regard to ecosystem health, with population urbanization having the most pronounced influence at the initial stages and land urbanization becoming the main driver affecting ecosystem health during the period of rapid development and transition. The interaction of population and land urbanization had a prominent impact on the ecosystem health of the GBA. Therefore, for cities or urban agglomerations being built in the Belt and Road region, paying attention to the effects of urbanization subsystems on ecosystem health; ensuring a proper combination of different elements, such as population, the economy, and land space allocation; and paying attention to urban ecosystem health are prerequisites for sustainable development;
(4) The urban junction central core has become the region where urbanization and ecosystem health interact most strongly. The effects of urbanization and ecosystem health impacts at the Guangzhou–Foshan and Guangzhou–Dongguan–Foshan urban junctions are spatially evident. In the future, we need to be alert to ecological problems at urban junctions for cities along the “Belt and Road” in order to avoid damage to ecosystem health in the process of integrated urban development.

## Figures and Tables

**Table 1:** Study data sources.

Data Types	Data Name	Spatial Resolution	Sources	Website
Physical geographical data	Land-use data	30 m	GlobeLand30: Global Geo-information Public Product	http://www.globallandcover.com, (accessed on 7 July 2022)
	GDP data	1 km	Resource and Environmental Science and Data Center	https://www.resdc.cn/, (accessed on 15 July 2022)
	POP data			
	DEM data	30 m	Resource and Environmental Science and Data Center	
	NPP data	500 m	Production Gap-Filled Yearly L4 Global 500 m SIN Grid	https://lpdaac.usgs.gov/products/mod17a3hgfv006/, (accessed on 18 July 2022)
Socioeconomic data	Grain production and sown area	/	Guangdong Statistical Yearbook of 2000–2020	http://www.stats.gov.cn/tjsj/ndsj/, (accessed on 20 July 2022)
	Grain prices	/	National compilation of agricultural cost–benefit information	/

**Table 2:** The ecosystem resilience coefficients (ERCs) of land-use types in the GBA.

Type of Land Use	Cultivated Land	Forest Land	Grassland	Water Areas	Urban Land	Unused Land
ERC	0.4	0.8	0.65	0.8	0.2	1

**Table 3:** The ESVs of the different ecological system unit areas in the GBA (CNY/hm²·a).

Service	Cultivated Land	Forest Land	Grassland	Water Area	Urban Land	Unused Land
Food production	2877.54	1734.99	1481.09	1692.67	0	0
Raw material production	190.43	3998.94	2179.32	486.64	0	0
Water supply	−5564.66	2073.52	1206.03	17,540.31	0	0
Gas regulation	2348.58	13,160.52	7659.34	1629.2	0	42.32
Climate regulation	1206.03	39,354.62	20,248.59	4845.27	0	0
Environmental purification	359.69	11,446.69	6686.05	11,742.91	0	211.58
Hydrological regulation	5755.28	24,522.59	14,832.04	216,323.48	0	63.48
Soil conservation	21.16	16,016.91	9330.85	1967.73	0	42.32
Maintenance of nutrient circulation	402.01	1227.19	719.39	148.11	0	0
Maintenance of biodiversity	444.33	14,578.14	8484.52	5395.39	0	42.32
Provision of aesthetic landscapes	190.43	6389.84	3745.04	3998.94	0	21.16

**Table 4:** Percentage area and relative change in EHI values in the GBA, 2000–2020.

Ecosystem Health Rating	2000	2010	2020	2000–2010	2010–2020	2000–2020
Weak	0.78%	1.46%	3.26%	0.69%	1.79%	2.48%
Relatively weak	12.19%	11.30%	14.91%	−0.89%	3.61%	2.72%
Ordinary	24.27%	22.40%	24.31%	−1.88%	1.91%	0.04%
Relatively good	35.90%	43.85%	48.93%	7.96%	5.08%	13.03%
Good	26.86%	20.99%	8.59%	−5.88%	−12.40%	−18.27%

**Table 5:** Estimation parameters for GWR models.

Year	Parameters				
	Residual Squares	Sigma	AICc	R²	Adjusted R²
2000	313.766	0.404	3714.590	0.879	0.836
2010	346.015	0.427	4028.207	0.867	0.818
2020	372.824	0.410	3215.343	0.856	0.832

**Table 6:** Detection of the impacts of urbanization subsystem factors on ecosystem health, 2000–2020.

q-Value	2000	2010	2020
X_1_	0.331	0.319	0.306
X_2_	0.329	0.322	0.263
X_3_	0.201	0.208	0.342

**Table 7:** Percentages of different types of LISA clusters, 2000–2020.

Type	2000	2010	2020
Low–high	25.17%	25.29%	26.60%
High–low	8.67%	8.87%	12.84%
High–high	0.42%	0.58%	0.62%
Low–low	9.25%	8.10%	6.25%
Not significant	56.48%	57.17%	53.70%

## Data Availability

The data that support the findings of this study are available from the corresponding author upon reasonable request.
